# Accessing support and empowerment online: The experiences of individuals with diabetes

**DOI:** 10.1111/hex.12552

**Published:** 2017-07-18

**Authors:** Ellen Brady, Julia Segar, Caroline Sanders

**Affiliations:** ^1^ Centre for Primary Care University of Manchester Manchester UK; ^2^ NIHR School for Primary Care Research Manchester Academic Health Sciences Centre University of Manchester Manchester UK

**Keywords:** diabetes, online support, patient communities, patient empowerment, self management

## Abstract

**Context:**

The use of the internet for health information by those with long‐term conditions is growing. It has been argued that this represents a form of empowerment by patients, as it enables them to control the content and flow of the information available to them. To explore this, the use of online discussion groups by those with diabetes was examined.

**Method:**

Semi‐structured interviews were conducted with 21 participants with type 1 and 2 diabetes and analysed using thematic analysis. Participants were recruited via online and offline routes, namely discussion boards, newsletters, and research networks related to diabetes.

**Results:**

By drawing on the advice, information, and support shared online, participants were empowered to position themselves as active participants in their own health care and to further engage with health‐care professionals.

**Conclusion:**

The findings indicate that forums can play a valuable role in aiding and motivating individuals in the daily management diabetes and highlight how this support is used to complement formal health services. However, more work needs to be carried out to determine to explore when and under what circumstances online support may be particularly beneficial to those with long‐term conditions.

## 1 INTRODUCTION

While there has been considerable progress in the control and treatment of symptoms and effects for some conditions, people are rarely completely cured of long‐term conditions (LTCs).^1^ Given this, it can be difficult to meet the multitude of needs of chronically ill individuals within current health‐care settings. In an attempt to offer an improved method of managing LTCs, in addition to lessening demand on health services,^2^ there has been a recent policy shift in terms of the responsibility taken by individuals for their own health care.^3^ Accordingly, people are beginning to be encouraged proactively to seek out health information, advice, and support for their condition.^4^

As a result, individuals with LTCs are repositioned from passive recipients of expert care^5^ to active consumers who make informed choices and share responsibility for their health care.^6^ Although this is not to suggest that this behaviour did not always occur, it does indicate that the sanctioned role and identity of patients within the health system has shifted from a recipient of care within a paternalistic doctor–patient relationship to actively negotiating and shaping their care. In addition, changes in technology and internet use over the last few decades have meant that individuals’ sources of health information and advice have increased.^7^ This paper will focus on a specific source of health information for those with LTCs, namely online discussion groups.

Online discussion groups function by enabling individuals to engage in supportive interactions through forums, chat rooms, and individual email exchanges with others facing similar experiences, challenges, or problems.^8^ The benefits of computer‐mediated support groups include the potential to access support 24 hours a day, 7 days a week at a time most convenient to the user accessing support. This results in issues relating to family or work commitments becoming less problematic, while individuals who have difficulties with mobility, speech, and hearing experience less barriers to involvement.^9^

Alongside the psychological impact of online support groups for people with LTCs, there are a number of sociological aspects to consider. Notably, Bury's^10^ work around biographical disruption, where the onset of an LTC represents a disruption to an individual's personal narrative, needs to be explored. Similarly, Charmaz^11^ highlights the “loss of self” experienced by many with chronic illnesses, as the experiences and meanings upon which their identities are built are no longer available to them. She argues that this process can be complex and overlapping, with individuals’ restricted lives resulting in them withdrawing from social activities. This can in turn heighten their sense of a loss of identity due to the absence of opportunities for self‐validation through socializing with others.^12^

Addressing this notion, Hardey^13^ suggests that the array of information and advice available on the internet enables users to develop and redevelop their identity in a way that goes beyond the concept of a patient as a disembodied medicalized case.^5^ Due to the ability of internet users to browse and search online, individuals control the content and flow of the information available to them, rather than receiving information through a health professional. This represents a shift in hierarchy from professionals towards patients^13^ and indicates that the use of online support groups can facilitate the development of a person's illness identity.

This suggests that the internet could act as a valuable tool in reconstructing identities^14^ and repairing the loss of the self for those with LTCs. Individuals’ identities can be reconstructed from their former narratives into that of an active, empowered patient via interactions with other forum users online^15^ and access to specialized information and advice.^13^ While not looking directly at LTCs, Trondsen and Tjora^16^ reported that for adolescents with parents with mental illnesses, communicating via an online forum allowed them to normalize their experiences and to reorient and reconstruct their identity. Accessing a community based on shared experiences enabled the teenagers to adjust their identity and that of their family from “abnormal” or “mentally ill” to “less out‐of‐the‐ordinary.” Through this normalization, participants gained agency and were empowered to take action.

This has been echoed by a number of researchers (eg Barker^15^; Pitts^17^) who suggest that the autonomous nature of internet use empowers individuals through a number of strategies. Online discussion groups allow individuals to access a collective pool of information, which can benefit their health. In addition, they can access a network of social support borne from shared experiences. Taken together, these benefits of forum participation facilitate empowerment, which aids individuals in taking a proactive approach to their health‐care decisions.^18^, ^19^ In short, online support appears to empower lay individuals to engage with their health care, as well as consolidating their position as challengers to the concept of medical dominance.^13^ To explore this process further, the use of internet forums by individuals with LTCs, namely type 1 and type 2 diabetes, was examined.

The World Health Organisation has defined diabetes as “a chronic disease that occurs either when the pancreas does not produce enough insulin, or when the body cannot effectively use the insulin it produces” (WHO, p. 1).^20^ There are two main forms of diabetes: type 1, which is also known as insulin dependent or childhood onset diabetes, and type 2, which is known as non‐insulin dependent. Type 1 diabetes is characterized by a lack of insulin production in the pancreas, while type 2 diabetes is caused by inefficient use of insulin in the body and has been associated with obesity and a sedentary lifestyle.^21^

Diabetes affects more than 5% of the British population^21^ and has been highlighted by the NHS as a key focus of the effort to improve chronic disease management in the UK.^22^ Diagnosis involves a simple and routine blood test. In addition, it is one of the long‐term conditions included in the UK's Quality and Outcomes Framework (QOF), whereby clinicians are incentivized to provide evidence‐based care to individuals.^23^ This is not to suggest that living with diabetes is a simple process, however. There is an increasing stigma associated with the condition, with individuals using terms like “fat, lazy, unhealthy” to describe those with diabetes (Vishwanath,^24^ p. 523). This stigma impacts on people's self‐management of their diabetes, including their monitoring of their blood glucose levels.^25^ In addition, the progressive and chronic nature of type 2 diabetes means that the necessity for substantial lifestyle changes can place a heavy burden on individuals, their families, and health services.^26^

Regarding research around diabetes and online peer support, it has been suggested that diabetes forums contain broadly accurate health advice and information.^27^, ^28^, ^29^ In addition, the functions of the discussion groups appeared to replicate those found in other LTCs, with users seeking support, experiential knowledge, and information about medication and condition management, such as diet and weight control,^30^ and accessing interpersonal and community support along with specialized knowledge from peers.^29^ Similarly, Ravert, Hancock and Ingersoll^31^ reported that adolescents with type 1 diabetes visit internet forums for social support, advice, information, and access to shared experiences. This indicates that individuals access diabetes forums in line with other chronic conditions. As a result, this condition offers an appropriate context to shed further light on the use of the internet by individuals with LTCs.

## 2 METHODS

This research with individuals with diabetes took place as part of a broader piece of research on the use of forums by UK‐based individuals with LTCs.^32^, ^33^ A qualitative approach was selected as it allowed for an exploration of the opinions and perspectives of individuals with diabetes. Semi‐structured interviews enabled participants to discuss the topics that were relevant to their health and use of internet forums, rather than using a more prescriptive method of data collection. A broad interview schedule addressing participants’ use of diabetes forums was developed, although it should be emphasized that the schedule provided a guide for the interview and did not represent a prescriptive itinerary. Interviewees were given space to express their own opinions and ideas, and, in many cases, their responses shaped the order and structure of the interview.^34^

### 2.1 Participants

Twenty‐one participants completed interviews, 12 with type 1 diabetes and 9 with type 2 diabetes. Interviewees were drawn from across the UK, and the majority of the respondents were female (n=13), with a mean age of 52 (age range=20‐82 years). To ensure that a range of perspectives was considered, recruitment took place both online and offline. Interviewees were recruited through online and offline sites related to diabetes, such as internet forums, magazines, email lists, and research networks. Participants were offered the option of face‐to‐face or phone interviews with the researcher; many (n*=*17) chose to participate by phone. All interviewees described themselves as white.

### 2.2 Data analysis

Interviews were recorded and transcribed verbatim, with any potentially identifying information removed. The anonymized interview transcripts were imported into a qualitative data analysis computer software package, ATLAS.ti version 7, to carry out the analysis. It should be noted that the use of a software package merely provided a tool to organize and review the data during the analysis process, rather than offering an objective method of analysis.^35^ Using an iterative process, each transcript was read through several times and notes were made to make note of preliminary connections between interviewees.

A thematic method of analysis was employed, with a view to examining comparisons and contrasts across participants and within cases. Thematic analysis was chosen as it provided a flexible approach to analysing qualitative data and involves identifying themes in a body of data.^36^ Themes were considered to capture something important about the data, and to represent a level of patterned response or meaning within a data set. This process allows the development of a conceptual scheme which enables the researcher to ask questions of the data.^37^ The initial themes that had been noted throughout the initial analysis process provided the basis for an exploratory coding frame, where labels were assigned to portions of the transcripts. This served as a process of “data reduction,” whereby the boundaries of analysis are delineated (Namey, Guest, Thairu, & Johnson^38^).

The data were coded according to these themes by identifying complete segments (eg sentences or paragraphs) as categories that had been isolated and defined during the course of the research. Initially, these codes were broadly descriptive, and related directly to the content of interviewees’ transcripts, rather than any more subtle nuances within the data. For example, references to an interviewee's family were coded as “Family,” and so on. Following this initial coding attempt, the themes and transcripts were reviewed and discussed amongst all three authors to ensure the validity of the coding frame.

As coding continued, categories were broken down into further sub‐categories, as the initial coding frame did not sufficiently capture the complexities of the data. For example, while many participants described the interactions that they had with other forum members (coded as “Forum”), a number also spoke specifically about the interactions that they had with forum moderators. Midway through the analysis process, an additional code of “Moderator” was created, as these interactions were distinct from those that interviewees had with normal posters and required further consideration. Transcripts were reviewed on an on‐going basis to ensure that additional codes were applied.

### 2.3 Ethics

Ethical approval was granted by the University of Manchester research ethics committee. Any identifying information was removed from the interview transcripts, and all participants have been assigned pseudonyms. Each participant was provided with an information sheet and encouraged to contact the researcher with any questions both before and after the interview. Signed consent was received from all participants; for telephone interviews, the consent form was posted in advance along with a stamped addressed envelope to return the signed form to the researcher.

## 3 RESULTS

In this paper, how individuals draw on the support and shared experiences of other forum members to empower themselves and position themselves as active participants in their own health care will be detailed. Hardey^13^ suggests that the array of information and advice available on the internet enables users to develop and redevelop their identity in a way that goes beyond the concept of a patient as a disembodied medicalized case.^5^ By participating in forums and providing advice, information, and support to others, it will be argued that interviewees were able to further empower themselves as caregivers rather than just passive recipients of expert care. In this way, online support groups can facilitate the development of an individual's illness identity and restore a loss of self.^11^, ^15^, ^16^

### 3.1 Accessing support and shared experiences online

A number of interviewees described accessing health information online as an act of empowerment, something that supported them in their interactions with health‐care professionals and allowed them to negotiate with the medical profession.^13^ In this way, individuals armed themselves with information and were better able to have informed conversations and make decisions about their own health care and self‐management.^18^ Julie reported that using the internet for health information and advice had a positive impact on her confidence and her ability to actively engage with health‐care professionals. Rather than just passively receiving care, she felt that the information and support she had gained allowed her to participate in shared decision making and take an active role in managing her condition, “I'll be able to have a discussion about whether to reduce or maybe come off the metformin, and I feel confident that I'll be able to have a sensible conversation and that I will understand what I'm being told*”* (Julie, type 2 diabetes, 46–50).

Forums allowed participants to communicate with people who had a shared understanding of living with diabetes. This enabled forum users to access to a form of emotional support by venting to people with similar lived experiences. Venting meant that they could joke about their condition in a way that they were unable to do with their family and friends, who may worry or overreact, “And if I said that, to say, my mum, she'd be mortified…. Whereas other people can see the funny side of it. You can't really have that kind of gallows humour with somebody who doesn't get it” (Claire, type 1 diabetes, 41–45). The mention of “gallows humour” is particularly notable, as it has been suggested that using humour and jokes online can be viewed as a form of emotional support.^39^ This enabled users to discuss aspects of their condition in a light‐hearted manner, without having to explain the humour to someone who is not a member of the community. As a result of these types of communications, online communities often represented a place where individuals with LTCs could feel “normal” amongst their family and friends.^16^ Interviewees took solace in the knowledge that they were not alone in their experiences and that others were going through similar struggles, “Sometimes there is some sort of comfort just knowing that there's other people out there going through it” (Daniel, type 1 diabetes, 26–30).

This sense of shared experiences also meant that forums provided participants with information that they could not access via their health‐care professionals, notably the experiences of people who were similarly engaged in living with and managing diabetes. This allowed them access to a form of “patient knowledge,” where individuals translated medical knowledge into practical courses of action.^40^, ^41^ For those with diabetes, online discussion groups enabled them to receiving information from people who were facing similar challenges and issues on a daily basis, unlike health‐care professionals.

Crucially, this allowed participants to question forum members about their lived experiences with their condition. This was illustrated by Gemma, who described going to the doctor a few months after falling off a horse and injuring herself in a number of ways. While she was sure that her on‐going problems were related to these injuries rather than her type 2 diabetes, she felt that her doctor was unable to look past her diagnosis and consider other options, “I've got this label now which is diabetic and I feel like I'm only ever going to be, have something diabetic considered about me and not anything else” (Gemma, type 2 diabetes, 31–35). As a result, she looked for others who had experienced the “feeling” of neuropathy, as opposed to the qualifications of the doctor, who was just aware of the classification. Seeking support online allowed her to access the form of experiential information that she required,^42^ as well as providing her access to a community where she was seen as an individual rather than a condition.^43^

### 3.2 The internet as a tool of empowerment

The use of internet forums by individuals with diabetes appeared to be contextually dependent, with interviewees utilizing online sources for routine queries rather than acute situations,^44^, ^45^ and with a frequent deferral to the expertise of the medical community.^42^ For example, participants were often seen to share “tips and tricks” with other forum members, ie suggestions that were intended to support individuals to self‐manage their conditions. Typically, these were drawn from their own experiences of diabetes and related to minor inconveniences rather than major medical issues.

Illustrating this, Louise described how she had altered her night‐time routine as a result of advice that she received on a diabetes forum. While she used to keep a bottle of Lucozade by her bed to prevent her blood sugar becoming low during the night, she found that the size of the bottle meant that she would invariably drink too much, “The problem with that is that Lucozade comes in big bottles and the temptation is to drink a lot of it and then your blood sugar ends up too high” (Louise, type 1 diabetes, 31–35). Based on the experiences of others, she began to use small boxes of juice that were usually marketed at children, “They're just the right size to treat a low blood sugar in the middle of the night. So you can't take too much of it, and also they last for ages and you're not left with an open box”. As she had not conceptualized her use of Lucozade as problematic, it was not something that she sought help or advice on. However, interacting with other peers online provided her with practical knowledge about her condition which she was able to utilize in her daily—and nightly—practices.^40^, ^41^

Along with providing practical advice and support, accessing shared experiences online also often provided participants with motivation. For individuals who were trying to lose weight or to focus on a particular food plan, particularly those with type 2 diabetes, forums provided a location for users to support each other. Forum members shared advice, experiences, and information to motivate each other towards healthy behaviours. Participants were able to access non‐judgemental, personalized advice and support that was tailored towards their individual lifestyle and preferences, as demonstrated by Lesley.

We support one another and say, keep going, don't give up, you know, that kind of thing… You can actually break down somebody's menu and say, well, where are you sticking, what point of the day are you having difficulty with, what food group are you having difficulty with, let's find an alternative that's low carbohydrate, or diabetic friendly, shall I put it, which is also suitable for you as an individual. (Lesley, type 2 diabetes, 56–60)

Similarly, accessing online discussion groups upon her diagnosis allowed Gemma to engage with people who were making what she perceived to be healthy lifestyle choices. This provided her with both guidance in managing her type 2 diabetes, and optimism and motivation that she would be able to do so successfully. “These are people who have all got diabetes, they've all had to live with it for however many years and they're all actively trying to manage it as best they can…They try lots of different things and they tell you what does and what doesn't work for them and it's all ideas for me to try” (Gemma, type 2 diabetes, 31–35).

Accessing support online also empowered interviewees to engage not just with their own diabetes but also with formal health‐care systems. Laing et al.^43^ suggest that participating in a forum allows individuals to develop a strong sense of ownership over their condition, which in turn leads to them becoming more actively engaged with health‐care professionals. This was illustrated by Margaret, who described the difficulties that she had encountered in receiving appropriate treatment for her diabetes. Although she had initially been diagnosed with type 2 diabetes, she had experienced a number of difficulties with the medications that she had been prescribed, including regular hypoglycaemic episodes and very high blood sugar levels. This had prompted suggestions from posters on the forum that she used that she had LADA (latent autoimmune diabetes of adults) or “type 1.5″ due to her unsuccessful cycle of medications. This in turn led to her pushing for a prescribed course of multiple daily injections, which successfully lowered her blood glucose levels. Although her doctor was initially reluctant to acquiesce, Margaret was able to make a case for her preferred choice of medication, thereby placing herself as an active participant in her own health care.

Throughout this experience, she engaged in a process of negotiation and renegotiation, making multiple visits to her GP, while simultaneously consulting the forum, the manufactures of the medication and NICE guidelines. Although Margaret attributed her success in this “battle” to the support and encouragement from the forum, “Now if it hadn't have been for the forum, I wouldn't really have known how, what, where to go ahead with a situation like that” (Margaret, type 2 diabetes, 66–70), it is clear from her account that she was more than a passive recipient of support. Instead, she engaged with and built on the support, information, and advice provided online, using it to explore and research the different options available to her. This allowed her to take an active approach to her health‐care decisions.^18^

However, her frequent engagement with her doctor indicates that her online activity did not signal a rejection of the medical profession.^46^ Instead, Margaret used the internet as a tool of empowerment, drawing on her own knowledge about her health and illness to complement and engage with the services offered in formal health‐care settings.^47^, ^48^ Indeed, many of the interviewees reported that their doctors did not fully appreciate the empowering role that the internet played in supporting them to manage their diabetes, “I don't think enough people are getting the benefit from them [forums], and I think there is a lot of work we need to do around helping healthcare professionals understand the potential benefits” (Louise, type 1 diabetes, 31–35).

### 3.3 The value of reciprocal support online

In addition to receiving support online, the reciprocal nature of forums and other forms of online communication meant that participants were able to provide support as well as receive it. It has been suggested that online discussion groups can fulfil a dual purpose: providing information to those who need it, as well as allowing those offering advice, information, and support to others to feel that they play a positive and useful role.^49^, ^50^ While many participants provided support to others online, there were suggestions that they themselves benefited from the provision of support. For June, who had been using diabetes forums for several years, responding to people's questions and queries online meant that her own knowledge base about her diabetes had expanded, “I have learnt an awful lot because if somebody asks a question and I don't know the answer to it, I'll go and research it so I can give them an answer. And I think that does sort of help me” (June, type 1 diabetes, 66–70).

This indicates that patient empowerment online can extend beyond an individual's engagement with their own condition. By interacting with peers and providing them with support, forum users were able to mobilize their existing capabilities on behalf of others. Providing support to others with diabetes allowed forum members to engage in reciprocally supportive relationships, in contrast to the concept of patients as passive recipients of expert care.

## 4 DISCUSSION

The findings showed that internet forums acted as a tool of empowerment, allowing people to draw on the shared experiences of others to position themselves as active participants in their own health care and to distance themselves from the traditional sick role.^5^, ^19^, ^51^ In this way, interviewees armed themselves with information and were better able to have informed conversations and make decisions about their condition.^18^ Crucially, this support empowered individuals to engage with health‐care services and to be proactive about their own health and the self‐management of their condition. This indicates that online forums offered an alternative yet complementary venue for forum users to access health‐related advice, information, and support, rather than a rejection of the medical profession.^46^, ^52^, ^53^, ^54^

Interviewees described receiving comfort and support from the knowledge that there were others in similar situations, as well as benefitting from the lived experiences of their peers. In this way, the use of online discussion groups can be likened to the creation of patient knowledge.^41^ Forum members shared “tips and tricks” online, enabling those accessing forums to learn a form of practical knowledge that was distinct from that offered by health‐care professionals. Just as Pols^40^, ^41^ described the knowledge sharing that occurred between individuals with chronic obstructive pulmonary disease, whereby they learnt to live with their condition by developing and sharing contextual, embodied knowledge, forum members drew on fellow patients’ daily lives to transform medical knowledge into techniques that individuals could use to manage their diabetes. Access to this form of experiential knowledge meant that forum users were provided with a more personalized form of support than could be accessed through health‐care professionals. This enabled them to assess and understand their health and health care in the context of other forum members’ experiences.^55^

Interestingly, online forums appeared to offer not just support and information but also motivation to engage in healthy behaviours, suggesting that online support can lead to changes in health behaviour. It has been reported that accessing support from one's offline network is associated with higher levels of motivation and engagement in health promoting behaviours,^56^, ^57^, ^58^ while Ayers and Kronenfeld^52^ found that the more an individual uses the internet for health information, the more likely their health behaviour is to change. Laing, Keeling, and Newholm^43^ suggest that participating in a forum allows individuals to develop a strong sense of ownership over their condition, which in turn leads to them becoming more actively engaged with health‐care professionals.

In addition, it is also possible that documenting one's behaviour online provides individuals with a sense of accountability. Describing a project where participants shared photographs online of their healthy meals, Parker^59^ reported that those sharing photos took care to ensure not just that their meals were nutritionally balanced but that they were presented in such a way that they appeared appealing to others. She suggested that as participants modelled healthy behaviours for others, they became motivated to live up to their own positive example. In other words, this dual process of inspiring others to eat healthily as well as monitoring their own eating behaviours resulted in benefits for individuals as well as the community in which they were active. This is echoed in this paper, where interviewees described detailed discussions around weight loss and healthy eating, where they monitored their own behaviour while simultaneously making health choices. This is particularly noteworthy given the negative reactions of many in the medical community to the rise of patients accessing health information online,^48^, ^60^ as mentioned by interviewees. Given this, it is necessary to reflect on the role that health‐care professionals could play in the provision of online support and how this role could be optimized to ensure that benefits for those with LTCs prevail.

Participants valued being able to share their experiences and provide support to others, and described the personal gain that they received from this process. These mutually supportive relationships allow individuals to overhaul their role from one in which they merely received instruction and information from health‐care professionals. In this way, forum users were further facilitated to distance themselves from the role of the passive patient^5^ and were instead able to position themselves as caregivers as well as receivers, adding to their sense of self‐worth. While this notion has received less attention in the literature, these findings are in line with previous research which suggested that forums allow those providing support and advice to feel valuable and useful.^49^, ^50^, ^61^ Supporting this argument, Mo and Coulson^62^ suggest that supporting others and sharing stories online helps forum users to understand what has happened to them and to let go of the past. It provides additional evidence of forums facilitating the narrative reconstruction of individuals’ identities^14^ and addressing the sense of loss of self following the onset of a chronic condition.^11^, ^63^ Indeed, it echoes the concept of illness gains described by Asbring,^64^ where individuals with LTCs experience new insights and an adjustment in priorities as a result of their condition.

Sharing experiences online allows individuals with diabetes to reposition themselves as active, empowered, and motivated patients who successfully engage in healthy behaviours.^15^, ^59^ This indicates that the use of internet forums can address the biographical disruption^10^ and the loss of self experienced following the onset of an LTC.^11^ As a result, forum users are facilitated in developing a new identity—one which differs from the passive sick role construct described by Parsons,^5^ but one which is also distinct from an individual's offline persona. Sharing experiences with others online provided interviewees with an opportunity to engage in mutually supportive relationships in which they could mobilize the resources available to them to assist others^49^, ^61^, ^62^ and which offered them an opportunity to discuss topics that they were not able to share with family and friends.^16^, ^65^

Finally, it should be acknowledged that online support may have a particular resonance for different individuals at different points during their illness trajectory. For example, in the stages after a diagnosis, support which is directed at regulating one's emotions may be particularly important.^66^, ^67^ Charmaz^63^ highlights the cyclical nature of adaption to the diagnosis of a chronic illness, suggesting that individuals’ needs do not remain consistent over time and may fluctuate depending on their own situation and that of their condition. As a result, the services and supports that people access will likely change over time. Similarly, Synnot et al. 68 reported that, for individuals with MS, their information needs, emotional stages, and expertise about their condition varied continuously. As a result, the usefulness of the internet as a resource also fluctuated. Consideration of the potential role of online support in the lives of those with LTCs should be viewed with this fluctuation in mind. In addition, this is a potential area of further research, to explore when and under what circumstances online support may be particularly beneficial. This information could guide health‐care professionals in their interactions with individuals with LTCs, and may be similarly useful for forum users and moderators.

In conclusion, the results indicate that forums play a valuable role in empowering individuals with diabetes and supporting them to take an active role in managing their condition. However, more research is required on the stages of adaption to an LTC and how online support can facilitate this adaption, as well as how to signpost forums so that individuals with LTCs can expediently access timely and relevant support and information online.
